# Prevalence and Impact of BRAF mutation in patients with concomitant papillary thyroid carcinoma and Hashimoto’s thyroiditis: a systematic review with meta-analysis

**DOI:** 10.3389/fendo.2023.1273498

**Published:** 2023-11-17

**Authors:** Lukasz Janicki, Agastya Patel, Jarosław Jendrzejewski, Andrzej Hellmann

**Affiliations:** ^1^ Department of Surgical Oncology, Medical University of Gdansk, Gdansk, Poland; ^2^ Department of General, Endocrine, and Transplant Surgery, Medical University of Gdansk, Gdansk, Poland; ^3^ Regional Hepato-Pancreato-Biliary Surgical Unit, Manchester Royal Infirmary, Manchester, United Kingdom; ^4^ Department of Endocrinology and Internal Medicine, Medical University of Gdansk, Gdansk, Poland

**Keywords:** Hashimoto thyroiditis, papillary thyroid carcinoma, BRAF mutation, lymph node metastasis, extrathyroidal extension, multifocality

## Abstract

**Background:**

Evidence suggests that patients with Hashimoto thyroiditis (HT) are at significantly higher risk of developing papillary thyroid cancer (PTC). However, the course of PTC in patients with both diseases concomitantly has been found to be more indolent than conventional PTC. Additionally, it has been well proven that BRAF mutation results in an aggressive course of PTC. The aims of this meta-analysis were to identify prevalence of BRAF mutation and its impact on clinicopathological features in patients with concomitant PTC-HT.

**Methods:**

Medline, Cochrane Library, Scopus, and Web of Science were searched until 16.09.2022, resulting in 227 articles, of which nine studies were included. Summary estimates, comparing patients with (A) BRAF (+) PTC-HT versus BRAF (+) PTC, and (B) BRAF (+) PTC-HT versus BRAF (-) PTC-HT, were generated with Review Manager 5.0.

**Results:**

In total, 6395 patients were included in this review. PTC-HT patients had significantly less BRAF mutation than PTC patients (Odds Ratio (OR) (95% Confidence Interval (CI))=0.45 (0.35-0.58), P<0.001). BRAF (+) PTC-HT patients were significantly more likely to have multifocal lesions (OR (95% CI)=1.22 (1.04-1.44), P=0.01) but less likely to have lymph node metastasis (OR (95% CI)=0.65 (0.46-0.91), P=0.01) and extrathyroidal extension (OR (95% CI)=0.55 (0.32-0.96), P=0.03) compared to BRAF (+) PTC patients. BRAF (+) PTC-HT patients were more likely to have multifocal lesions (OR (95% CI)=0.71 (0.53-0.95), P=0.02), lymph node metastasis (OR (95% CI)=0.59 (0.44-0.78), P<0.001) and extrathyroidal extension (OR (95% CI)=0.72 (0.56-0.92), P=0.01) compared to BRAF (-) PTC-HT patients.

**Conclusion:**

This meta-analysis highlights that the lower prevalence of BRAF mutation in patients with PTC-HT than conventional PTC may explain the indolent clinicopathological course in this cohort.

## 1 Introduction

Papillary thyroid carcinoma (PTC) is the most common type of thyroid cancer, with increasing incidence globally (1). Despite its malignant nature, patients with PTC tend to have excellent prognosis, with 10-year survival rate of approximately 90% (2–4). Hashimoto’s thyroiditis (HT) is an autoimmune inflammatory disease characterized by thyroid-specific autoantibodies [anti-thyroglobulin (Tg) and anti-thyroid peroxidase (TPO)] and T-cells infiltration of thyroid gland (5).

Decreased levels of T_3_ and T_4_ seen in hypothyroidism lead to an increase in thyroid-stimulating hormone (TSH) production, which in turn increases the risk of PTC development (6). Recent evidence suggests that, despite contributing to PTC development by causing hypothyroidism, HT may also ameliorate its malignant potential (1). It is suspected that the T-cells infiltrating thyroid tissues in HT secrete IL-1 responsible for inhibiting tumor cell growth and progression (7). Therefore, it is possible that patients with PTC and HT have favorable tumor characteristics (such as less lymph node metastasis (LNM), extrathyroidal extension (ETE), and decreased capsular and angio-invasion), and thereby better prognosis compared to those without HT (2, 8, 9). As HT has the potential to induce PTC, but also mitigate its progression, it can be referred to as a “double-edged sword” in relation to PTC (10).

The BRAF gene encodes a protein involved in the mitogen-activated protein kinases (MAPK) signaling pathway. BRAF mutation is a single nucleotide substitution, which results in continuous activation and signal transduction through the MAPK pathway, resulting in increased cell proliferation and differentiation (11). It is well known that mutation in the BRAF gene plays a crucial role in development of PTC (12). It is present in approximately 44% of PTC patients making it one of the most common somatic alterations in this type of cancer. An imperfect, but nevertheless a viable diagnostic marker (12). BRAF mutation in PTC has been associated with more aggressive clinicopathological features and outcomes such as LNM, ETE and overall mortality (13–15). Evidence also suggests that BRAF mutation in HT patients is associated with development of PTC and more aggressive clinical course (11, 16).

The aim of this meta-analysis is to identify the prevalence of BRAF mutation among patients with concomitant HT and PTC versus conventional PTC patients. The secondary aim is to evaluate the impact of BRAF mutation and HT on clinicopathological features in patients with PTC.

## 2 Methods

### 2.1 Literature search

This systematic review was conducted in accordance with the PRISMA (Preferred Reporting Items for Systematic Reviews and Meta-Analyses) statement using PICO (patients, interventions, comparisons, outcomes)-based questions. After formulating an appropriate search strategy, PubMed, Cochrane, Web of Science, and Scopus online databases were searched for suitable articles. No filters were applied during the search. Backward citation chaining of the chosen full-text studies was performed as well.

### 2.2 Evidence acquisition

**Figure 1:**
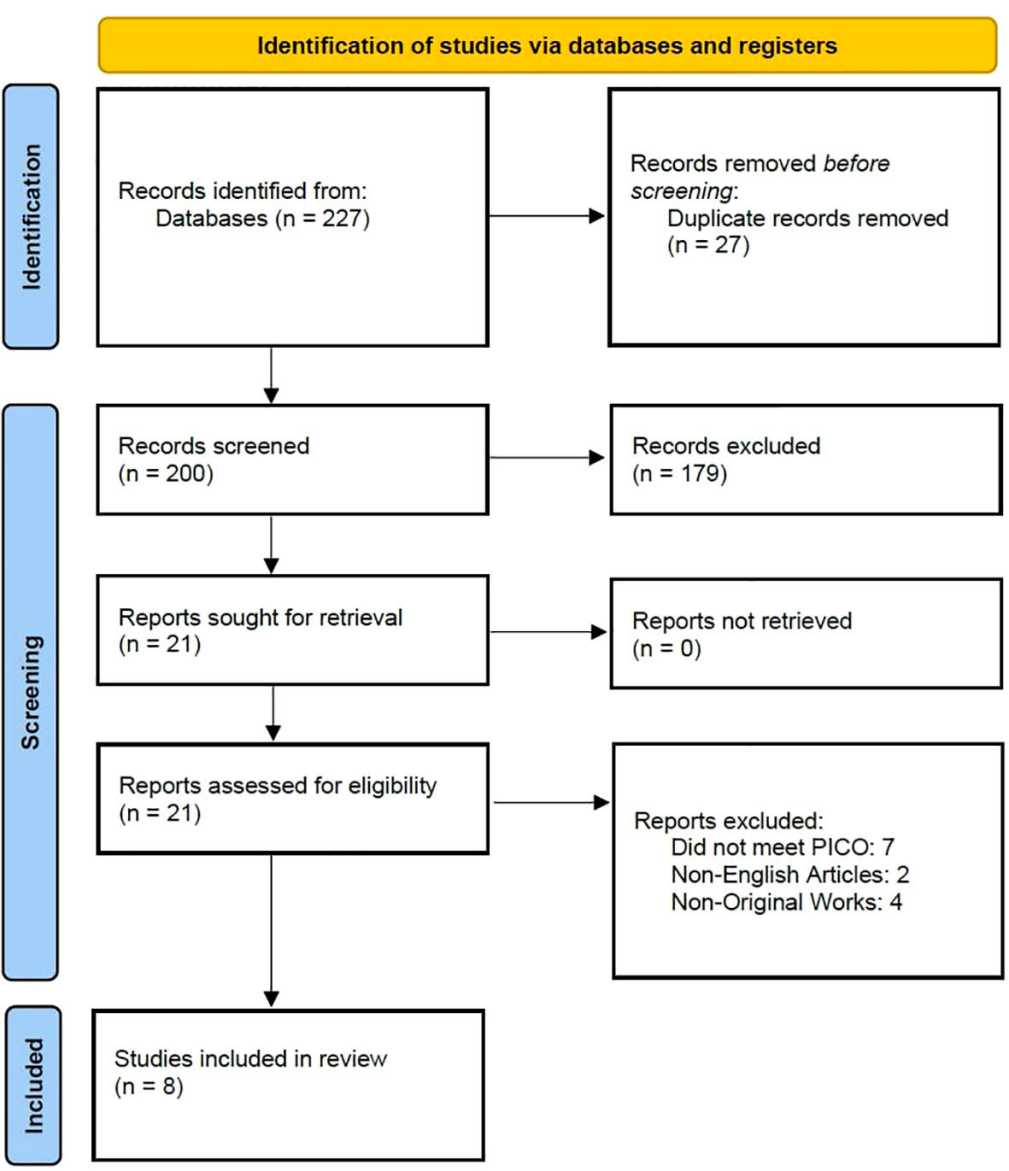
PRISMA flowchart.

On September 16^th^, 2022, two independent researchers (LJ and AP) searched the previously mentioned databases for suitable studies. The search string used was (“thyroid cancer” OR “thyroid carcinoma” OR “thyroid microcarcinoma”) AND (“BRAF” OR “mutation”) AND (“Hashimoto thyroiditis” OR “chronic lymphocytic thyroiditis” OR “Hashimoto’s thyroiditis”). The preliminary search, after deleting duplicates, resulted in 227 articles which were later screened by two independent researchers (LJ and AP). The entire process is presented in a PRISMA flow chart (**Figure 1**).

### 2.3 Inclusion and exclusion criteria

PICO framework-based research questions were used for this review. Studies were included if they were full-text, original reports comparing BRAF mutation rates in patients with concomitant PTC-HT with conventional PTC. Additionally, studies were excluded if full text was not available, was not in English, were not original articles or did not conform with PICO.

### 2.4 Evidence synthesis and quality assessment

Information from the eligible studies was retrieved by two independent researchers (LJ and AP) and summarized in tables. Any conflicts relating to inclusion of studies and collected data were resolved via discussion between the two authors in consultation with a third author (AH). Data on the following parameter was collected: first author, year and country of publication, inclusion and exclusion criteria, method of BRAF mutation analysis, demographic information (age, gender), prevalence of BRAF mutation and clinicopathological features.

The quality of cohort studies was assessed by two independent researchers (AP and LJ) using the Newcastle-Ottawa scale (NOS). A maximum of nine points for each of the following items was awarded: four stars for selection, two for comparability and three for outcomes. High quality studies scored seven or more stars on NOS assessment.

### 2.5 Statistical analysis

Weighted arithmetic means for the prevalence of BRAF mutation in PTC-HT and PTC was calculated by multiplying the weight of each study with the percentage of BRAF (+) patients in each group. The pooled summary estimates were created to compare the prevalence of BRAF mutation between the PTC-HT and PTC patients. Additionally, to assess the impact of BRAF mutation on clinicopathological features (multifocality, extrathyroidal extension (ETE) and lymph node metastasis (LNM), subgroup analyses were performed comparing (1) BRAF (+) PTC-HT versus BRAF (+) PTC patients and (2) BRAF (-) PTC-HT versus BRAF (+) PTC-HT patients. The pooled estimates were generated as odds ratio (OR) with 95% confidence interval (CI) using the Mantel-Haenszel (M-H) test. Random effects model was used if there was significant statistical heterogeneity measured using the I^2^ coefficient, otherwise the fixed effect model was utilized. The statistical analysis was performed with the Review Manager 5.4.1 software.

## 3 Results

After screening and full text assessment, nine articles (six retrospective, three prospective cohort studies) were included in this review. The information retrieved from the eligible studies is shown in **Table 1**. Across all studies, concomitant HT was diagnosed based on histopathological evaluation (diffuse lymphocytic infiltration, reactive germinal centers, parenchymal atrophy, stromal fibrosis) while three studies also used anti-Tg and anti-TPO antibodies (3, 15, 17). PTC was diagnosed based on histopathological analysis in seven studies (3, 4, 11, 15, 16, 18, 19) while two studies did not provide information on diagnostic methods (17, 20).

**Table 1:** Baseline characteristics of included studies.

Author	Study Design	PTC and HT Diagnosis	Grouping	N Patients	Age (Years)	Female (%)	BRAF (+) N (%)
**Kim et al., 2009, South Korea**	R	PTC: Histopathology HT: Histopathology	PTC	64	44.1 ± 13.4	84.4	61 (95.31)
			PTC-HT	37	49.4 ± 12.7	97.3	27 (72.97)
**Marotta et al., 2013, Italy**	P	PTC: Histopathology HT: Histopathology	PTC	92	56.1	71.7	72 (78.26)
			PTC-HT	54	50.2	92.6	44 (81.48)
**Kim et al., 2016, South Korea**	R	PTC: Histopathology HT: Histopathology	PTC	2326	47.6 ± 11.9	72.5	2015 (86.63)
			PTC-HT	1006	46 ± 11.4	90.7	774 (76.94)
**Zeng et al., 2016, China**	R	PTC: Histopathology HT: Histopathology	PTC	397	45.5 ± 11.8	72.8	325 (81.86)
			PTC-HT	222	45.9 ± 12.1	87.8	140 (63.06)
**Kim et al., 2018, South Korea**	R	PTC: NR HT: Histopathology	PTC	124	50.06 ± 11.51	86.3	102 (82.26)
			PTC-HT	48	46.44 ± 10.62	93.8	28 (58.33)
**Ozdamar et al., 2020, Turkey**	P	PTC: Cytopathology HT: Histopathology	PTC	19	45.68 ± 14.3	84.2	6 (31.58)
			PTC-HT	18	47.94 ± 14.84	100	5 (27.78)
**Kim et al., 2005, South Korea**	R	PTC: Histopathology HT: Histopathology	PTC	51	NR	NR	46 (90.20)
			PTC-HT	28	NR	NR	18 (64.29)
**Kim et al., 2016a, South Korea**	P	PTC: NR HT: Histopathology	PTC	1576	47.2 ± 12.0	81.8	1223 (77.60)
			PTC-HT	204	44.8 ± 11.9	97	122 (59.80)
**Issa et al., 2022, Switzerland**	R	PTC: NR HT: Sonography and Serology	PTC	106	NR	75.3	52 (49.06)
			PTC-HT	23	NR	85.2	9 (39.13)

R, retrospective; P, prospective; PTC, Papillary thyroid carcinoma; HT, Hashimoto thyroiditis; NR, not reported.

A total of 6395 patients were examined for BRAF mutation, of which 1640 had PTC concomitant with HT (PTC-HT) and 4755 had conventional PTC. BRAF mutation analysis in the included studies was performed using PCR amplification and DNA sequencing. Across studies, percentage of females ranged from 71.7 to 100% while median age ranged from 44.1 to 56.1 years. On risk of bias assessment, all studies were found to be of high-quality scoring >7 points on NOS.

### 3.1 Prevalence of BRAF mutation

**Figure 2:**
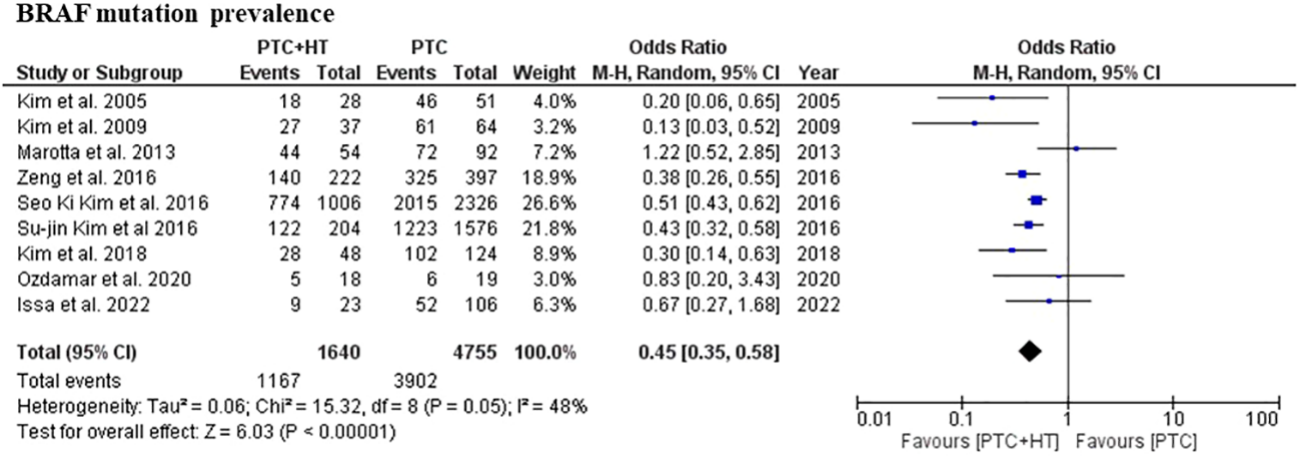
Summary estimate of BRAF prevalence in patients with PTC-HT versus conventional PTC.

All included studies reported the prevalence of BRAF mutation in patients with concomitant PTC-HT and PTC. The weighted arithmetic mean for BRAF mutation was 71.6% and 82.8% in PTC-HT and PTC patients, respectively. Patients with concomitant PTC-HT were found to have significantly lower prevalence of BRAF mutation than patients with PTC (OR (95% CI) = 0.45 (0.35-0.58), I^2^ = 54%, P<0.001) (**Figure 2**).

### 3.2 BRAF (+) PTC-HT versus BRAF (+) PTC

**Figure 3:**
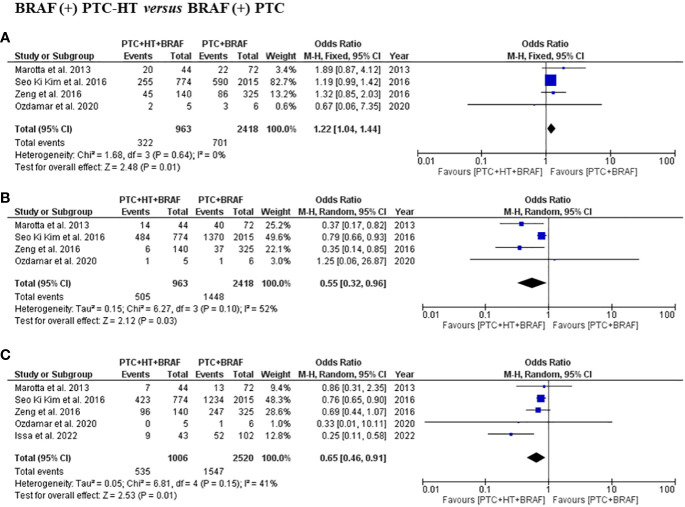
Summary estimates for **(A)** multifocality, **(B)** extrathyroidal extension and **(C)** lymph node metastasis comparing patients with BRAF (+) PTC-HT with BRAF (+) PTC.

Four studies presented information related to multifocality and ETE. In terms of multifocality and ETE, a total of 3381 patients with 963 patients in the BRAF (+) PTC-HT and 2418 patients in BRAF (+) PTC group were included (3, 15–17, 19). Patients with BRAF (+) PTC-HT were found to have significantly greater incidence of multifocal disease in comparison to BRAF (+) PTC (OR (95% CI) = 1.22 (1.04-1.44), I^2^ = 0%, P=0.01) (**Figure 3A**). On the other hand, the former group was found to have less ETE in comparison to BRAF (+) PTC (OR (95% CI) = 0.55 (0.32-0.96), I^2^ = 52%, P=0.03) (**Figure 3B**).

Data on LNM was available in five studies, with a total of 3526 patients with 1006 in the BRAF (+) PTC-HT and 2520 in BRAF (+) PTC group were included (3, 15–17, 19). This subgroup analysis showed that patients with BRAF (+) PTC-HT had significantly lower risk of developing central and lateral LNM than patients with BRAF (+) PTC (OR (95% CI) = 0.65 (0.46-0.91), I^2^ = 41%, P=0.01) (**Figure 3C**).

### 3.3 BRAF (-) PTC-HT versus BRAF (+) PTC-HT

**Figure 4:**
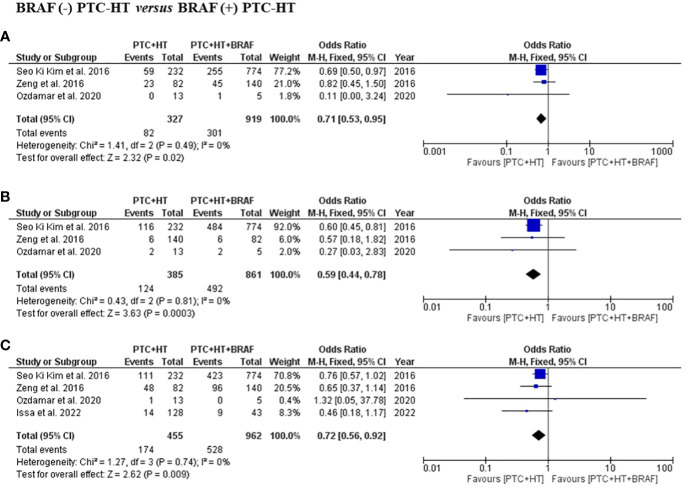
Summary estimates for **(A)** multifocality, **(B)** extrathyroidal extension and **(C)** lymph node metastasis comparing patients with BRAF (-) PTC-HT with BRAF (+) PTC-HT.

Three studies presented information related to multifocality and ETE. In terms of multifocality, a total of 1246 patients with 327 patients in the BRAF (-) PTC-HT and 919 patients in BRAF (+) PTC-HT group were included (3, 16, 19). BRAF (-) PTC-HT were found to have significantly less multifocal disease compared to BRAF (+) PTC-HT (OR (95% CI) = 0.71 (0.53-0.95), I^2^ = 0%, P=0.02) (**Figure 4A**). The same 3 studies provided data on 1246 patients in terms of ETE with 385 patients in the BRAF (-) PTC-HT and 861 in BRAF (+) PTC-HT group. The former group was found to have less occurrence of ETE (OR (95% CI) = 0.59 (0.44-0.78), I^2^ = 0%, P<0.001) (**Figure 4B**).

Information regarding LNM was available in four studies, with a total of 1417 patients with 455 in the BRAF (-) PTC-HT and 962 in BRAF (+) PTC-HT group (3, 16, 17, 19). This subgroup analysis showed that patients with BRAF (-) PTC-HT had significantly lower risk of developing central and lateral LNM compared to those with BRAF (+) PTC-HT (OR (95% CI) = 0.72 (0.56-0.92), I^2^ = 0%, P=0.01) (**Figure 4C**).

## 4 Discussion

To the best of our knowledge, this is the first meta-analysis reviewing the evidence on the relationship between BRAF mutation and concomitant PTC-HT. Most contemporary reviews have focused on the prevalence and clinicopathological features associated with concomitant PTC-HT. Previous systematic reviews have concluded that patients with HT are at increased risk of developing PTC, however, with a lower incidence of high-risk clinicopathological features (2, 10, 21). Over the recent years, increasing evidence suggested that BRAF mutation is associated with tumorigenesis and progression of PTC (22, 23). The BRAF mutation has been shown to be one of the strongest predictors for extrathyroidal invasion, lymph node metastasis and advanced tumor stages (24). Some have also proposed BRAF mutation scarcity among PTC-HT patients as an explanation for more indolent PTC course in this cohort (5). This review further investigates the above claims and demonstrates that patients with PTC and HT are significantly less likely to have BRAF mutation than those with conventional PTC.

The BRAF gene encodes a serine-threonine kinase, within the MAPK pathway, responsible for transferring signals from the extracellular matrix (ECM) to the nucleus. It regulates cell cycle, proliferation, growth, and survival. The BRAF mutation enables constitutive kinase activity, thus, elevating its activity almost 500-fold and making the MAPK continuously active (25). One of its actions in the nucleus is the methylation of tumor suppressing genes including tissue inhibitor of protein kinase 3, death-associated protein kinase, SLC5A8 and retinoic acid receptor β which are thought to be responsible for tumor inducing capabilities of BRAF (12). Moreover, PTCs harboring BRAF mutated alleles exhibit increased levels of MMP-2 and MMP-9 proteins, which break down ECM components as well as increase expression of vascular endothelial growth factor. These features play a role in BRAF-promoted PTC progression (12, 24, 26).

It is also postulated that the inherent characteristics of HT can facilitate development of PTC. One of the hypotheses points towards the increased production of thyroid stimulating hormone (TSH) as a consequence of HT-induced hypothyroidism. The elevated levels of TSH, a trophic hormone, may promote proliferation of thyroid follicular cells and increase the risk of tumorigenesis (6, 27, 28). Another theory suggests that diffuse lymphocytic infiltration associated with HT also leads to follicular cells dysregulation resulting in a tumor promoting effect (15). On the other hand, the presence of lymphocytic cells in the PTC-HT microenvironment increases the migration of myeloid-origin cells (Natural Killer-cells and macrophages), which are implicated in the late host-induced anti-tumor response (7, 15, 29). Ugolini et al. have demonstrated that anaplastic or poorly differentiated thyroid cancers, which have weaker lymphocytic infiltration, are associated with considerably poorer prognosis compared to PTC (30). Therefore, it appears that HT is a “promotor” of PTC, but may also simultaneously inhibit its progression. Conversely, some authors suggest the possibility that PTC arises first and triggers an immunological response against the thyroid gland resulting in HT (15, 17). Nonetheless, current evidence suggests a clear association between the two diseases, with HT having a protective effect towards PTC progression (1).

To investigate whether the protective effect of HT on PTC persists regardless of BRAF status, we analyzed the differences in clinicopathological features between BRAF (+) PTC, BRAF (+) PTC-HT and BRAF (-) PTC-HT. Firstly, our analysis demonstrated that patients with PTC-HT were 55% less likely to harbor BRAF mutation than PTC patients. Subsequently, BRAF (+) PTC-HT patients were found to have 45% and 35% less likelihood of ETE and LNM respectively compared to BRAF (+) PTC cohort. Similarly, BRAF (-) PTC-HT patients were 41% and 28% less likely to have ETE and LNM, respectively, than BRAF (+) PTC-HT patients. These findings confirm previous evidence suggesting a mitigating impact of HT on PTC, regardless of BRAF status (15). However, this effect may be slightly diminished in the presence of BRAF mutation. A potential explanation for lower LNM rates in PTC-HT patients may be due to the presence of HT-associated lymphadenopathy, resulting in more attentive monitoring and quicker PTC detection (15, 31). Furthermore, HT-induced inflammatory process involving IL-1, may be responsible for reduced ETE in PTC-HT patients (7, 31).

On the other hand, our analysis found an increased incidence of multifocal disease in patients with BRAF (+) PTC-HT compared to those with BRAF (+) PTC. These findings may be a result of varying cancerization processes. The increased levels of TSH implicated in HT-associated carcinogenesis influences follicular proliferation throughout the thyroid gland, resulting in greater number of lesions. Additionally, our analysis showed greater incidence of multifocal disease in patients with BRAF (+) PTC-HT than BRAF (-) PTC-HT. These findings are in line with the hypothesis presented by Lun et al. claiming that BRAF plays a key role in the TSH-involving cancerization process. As multifocality is not considered an aggravation feature, its increased incidence does not question the positive impact of HT on PTC (5, 21, 32).

Based on the results provided, we propose the foreknowledge of BRAF mutation status among PTC-HT patients can aid decisions on the extent of thyroid surgeries. It is known that inflammation associated with HT increases the risk of surgical complications (32). On the other hand, a recent randomized controlled trial has demonstrated that total thyroidectomy improves long-term health-related quality of life in patients with HT (33). In patients with BRAF (+) PTC-HT, total thyroidectomy should be favored, as per current guidelines, due to potentially more aggressive cancer course (34). However, in patients with BRAF (-) PTC-HT, a more conservative resection of the thyroid gland may be undertaken, in the absence of other high-risk features, to reduce the risk of complications. Therefore, it would be useful to assess BRAF mutation status as well as concomitant thyroid diseases while managing patients with PTC.

PTC microenvironment consists of a mixture of tumor cells with only a minority of them carrying BRAF. Therefore, it is plausible that BRAF mutation frequency in PTC-HT patients might be underestimated by the dilution of mutated cells caused by the wild type BRAF-containing lymphocytes. One of the limitations of our meta-analysis is that it is mainly based on retrospective studies. Some studies included a small number of patients, making it difficult to draw firm conclusions. Additionally, there was heterogeneity in reporting of PTC diagnostic criteria across studies. Moreover, majority of studies included in this meta-analysis originated from Asian countries, where the prevalence of BRAF mutation is known to be higher (22). Due to limited data on the BRAF-PTC-HT paradigm from Western countries, it is difficult to ascertain whether these findings translate into these patient cohorts. Therefore, there is a need for further prospective studies on the topic, including greater sample sizes, especially in the Western cohort.

## 5 Conclusion

Through our meta-analysis, we have shown that BRAF mutation occurs less frequently in patients with concomitant PTC-HT. BRAF (+) PTC-HT show greater chance of developing aggressive clinicopathological features compared to BRAF (-) PTC-HT, thus providing evidence that BRAF scarcity may be one of the factors responsible for the more indolent course of PTC in patients with concomitant HT. Based on the chosen clinicopathological features BRAF (+) PTC patients were more likely to develop poor prognosis than BRAF (+) PTC-HT confirming HT to be a protective factor against BRAF associated outcome.

## Data availability statement

The original contributions presented in the study are included in the article/supplementary material. Further inquiries can be directed to the corresponding author.

## Author contributions

LJ: Writing – original draft, Data curation, Formal analysis, Investigation. AP: Writing – original draft, Conceptualization, Data curation, Formal analysis, Investigation, Methodology, Project administration, Supervision, Visualization. JJ: Supervision, Formal analysis, Investigation, Writing – review & editing. AH: Writing – review & editing, Conceptualization, Investigation, Methodology, Project administration, Supervision.
